# Feel-Good Requirements: Neurophysiological and Psychological Design Criteria of Affective Touch for (Assistive) Robots

**DOI:** 10.3389/fnbot.2021.661207

**Published:** 2021-07-06

**Authors:** Mehmet Ege Cansev, Daniel Nordheimer, Elsa Andrea Kirchner, Philipp Beckerle

**Affiliations:** ^1^Chair of Autonomous Systems and Mechatronics, Department of Electrical Engineering, Faculty of Engineering, Friedrich-Alexander-Universität Erlangen-Nürnberg, Erlangen, Germany; ^2^Elastic Lightweight Robotics Group, Institute of Robotics Research, Department of Electrical and Information Engineering, Technische Universität Dortmund, Dortmund, Germany; ^3^Robotics Research Group, Mathematics and Computer Science, University of Bremen, Bremen, Germany; ^4^Robotics Innovation Center, German Research Center for Artificial Intelligence, Bremen, Germany; ^5^Institute for Mechatronic Systems, Department of Mechanical Engineering, Technical University of Darmstadt, Darmstadt, Germany

**Keywords:** affective touch, human-machine interfaces, tactile feedback, assistive robotics, design requirements

## Abstract

Previous research has shown the value of the sense of embodiment, i.e., being able to integrate objects into one's bodily self-representation, and its connection to (assistive) robotics. Especially, tactile interfaces seem essential to integrate assistive robots into one's body model. Beyond functional feedback, such as tactile force sensing, the human sense of touch comprises specialized nerves for affective signals, which transmit positive sensations during slow and low-force tactile stimulations. Since these signals are extremely relevant for body experience as well as social and emotional contacts but scarcely considered in recent assistive devices, this review provides a requirement analysis to consider affective touch in engineering design. By analyzing quantitative and qualitative information from engineering, cognitive psychology, and neuroscienctific research, requirements are gathered and structured. The resulting requirements comprise technical data such as desired motion or force/torque patterns and an evaluation of potential stimulation modalities as well as their relations to overall user experience, e.g., pleasantness and realism of the sensations. This review systematically considers the very specific characteristics of affective touch and the corresponding parts of the neural system to define design goals and criteria. Based on the analysis, design recommendations for interfaces mediating affective touch are derived. This includes a consideration of biological principles and human perception thresholds which are complemented by an analysis of technical possibilities. Finally, we outline which psychological factors can be satisfied by the mediation of affective touch to increase acceptance of assistive devices and outline demands for further research and development.

## 1. Introduction

Human-machine-interfaces and robots used for assistance, service or rehabilitation purposes currently exhibit very limited abilities to mediate subtle affective touch, i.e., tactile processing with a hedonic or motivational component (Morrison, 2016a), in humans (Beckerle et al., 2018). Conversely, they are not able to sense, process and understand such touch. In current robotics, tactile information is mostly used for precision grasps, which are mainly realized by intrinsic tactile sensing (Bicchi, 1990) with sensors placed within the structure of an end effector. Affective touch would require extrinsic tactile sensing via sensor arrays at the point of contact. In every application area where robots interact closely with humans, the ability to understand and use this level of tactile information can be advantageous (Beckerle et al., 2018). Interfaces mediating touch can profit from the ability to elicit affective feelings in their users, e.g., vivid haptic feedback can increase immersion in virtual or augmented reality applications (Hoffman, 1998; Ku et al., 2003). Moreover, psychological effects such as bodily illusions, where users get the impression that an artificial limb is their own one, benefit from such technologies (Crucianelli et al., 2013, 2018). It has been shown that affective information can enhance the rubber hand illusion and thus has the potential to create a more realistic experience of prostheses and other assistive devices (Crucianelli et al., 2013; van Stralen et al., 2014). Nevertheless, interfaces mediating affective information are scarcely researched and only few prototypic implementations exist (Bonanni et al., 2006; Huisman et al., 2013, 2016; Raisamo et al., 2013; Culbertson et al., 2018). Accordingly, further research is needed in order to improve and extend the capabilities of human-machine-interfaces and assistive robots with regard to affective tactile interactions.

For the purpose of designing appropriate technical devices, an understanding of the relevant neurobiological and psychological mechanisms in humans is required. Although current fundamental research of affective touch has not yet answered all open questions in those areas (Olausson et al., 2010), important progress has been made, which enables future technical development. This review summarizes key findings from neurobiological and psychological research to provide guidance for the design of future technical implementations. In section 2, we begin with a brief overview of known mechanoreceptors relevant to active touch, and then introduce the biological background of affective touch and its effects on psychological factors during human-machine interaction in section 3. Section 4 examines the previous implementations that aim for mediating affective sensations to offer design recommendations as section 5 concludes the review with a brief overview and discussion.

## 2. Neurobiological Background

In human glabrous and hairy skin, a large number of different sensory receptors are found in the dermis or epidermis. For example, nociceptors measure noxious mechanical or thermal events, and mechanoreceptors to measure tactile sensations. Different receptors and types of tactile fibers are known for the tactile innervation of glabrous and hairy skin. Some of them are common in both skin types, others differ and especially in hairy skin additional variations with respect to different parts of body were found (Vallbo et al., 1995). This goes along with differences in the function of glabrous and hairy skin. While hairy skin is more relevant for affective touch, which is the topic of this review, glabrous skin is more involved in discriminative touch, although, both types of skin are able to receive mediated touch as for example recently discussed in Corniani and Saal (2020). In order to transfer physiological findings to technical systems such as robots, sensing and feeling of the environment by receptors in the skin has been studied and characterized primarily with respect to the manipulation of objects (Johansson and Flanagan, 2009; Dahiya et al., 2010), i.e., active touch (Gibson, 1962). Hence, for defining design criteria for technical systems, myelinated Aβ afferents for discriminative touch, which are of higher density in glabrous skin, were in most cases considered (Corniani and Saal, 2020). In this review, we want to focus on affective or mediated touch and unmyelinated mechanoreceptors, which are highly related to affective touch, although it was recently suggested that both myelinated as well as unmyelinated fibers should be considered as rather interleaved than separated sources for different facets of tactile information (Marshall et al., 2019). For reasons of overview and differentiation, we first provide a brief overview of the sensory receptors in the human glabrous skin that are most relevant for tactile perception during object manipulation, i.e., that sense information for discriminative touch to be transferred by myelinated Aβ afferents, while briefly referring to differences between glabrous and hairy skin. Then, we focus on unmyelinated mechanoreceptors. Recently studied receptors which are hypothesized to play an important role in affective touch, i.e., C-tactile afferents, as well as brain processing of CT afferent signals are presented in more detail, to address the need for systematic reviews in this area (Corniani and Saal, 2020).

### 2.1. Mechanoreceptors for Discriminative Touch

For discriminative touch, four different types of myelinated Aβ afferents in different layers of the glabrous skin and with different distribution, morphology, and function (Vallbo and Johansson, 1984; Lederman and Browse, 1988) responding to mechanical pressure or distortion of the skin (Vallbo and Johansson, 1984) are mainly considered in design criteria for technical systems. In the following we give a brief overview on myelinated Aβ afferents of glabrous skin concentrating on their response characteristic and point out differences to hairy skin.

In glabrous skin, which is most relevant for the manipulation of objects, fast adapting (FA) tactile units responding to the transient phases of stimulation, i.e., responding only to changes in the signal, and slowly adapting (SA) units that are sensitive to static forces and show a sustained discharge can be differentiated (Vallbo and Johansson, 1984). These two main groups can further be differentiated, e.g., with respect to differences in responses to the stimulus pattern. For example, the SA sub-type SA1 units (Merkel corpuscle end-organs) are particularly sensitive to edge contours of objects (Johansson et al., 1982; Johansson and Vallbo, 1983). They show a higher response frequency during the beginning of contact and decreasing feedback over time (Johansson and Vallbo, 1983) while SA2 units (Ruffini corpuscle end-organ) are responding with a constant frequency during the contact phase (Vallbo and Johansson, 1984). Interestingly, SA2 units do not only respond to indentation but also to skin stretch with a directional property (Knibestöl and Vallbo, 1970; Johansson, 1976, 1978) and might therefore contribute to measure shearing forces (Vallbo and Johansson, 1984), e.g., caused by a tool slipping out of the hand. FA2 receptors (Pacinian corpuscle end-organs) respond particularly to rapid onset and offset of those (Johansson and Vallbo, 1983) and to high frequency vibrations (Johansson et al., 1982; Dahiya et al., 2010). In contrast, FA1 (Meissner corpuscle end-organ, Iggo and Muir, 1969) responds to rapidly occurring small changes in the indentation of the skin (Johansson and Vallbo, 1983), i.e., they respond as long as the stimulus is changing. FA1 receptors can only be found on glabrous skin unlike other aforementioned receptors that are also located on hairy skin (Vallbo et al., 1995). Rapidly adapting hair and field afferents replace F1 receptors in hairy skin (Vallbo et al., 1995) and might be more sensitive to higher frequencies (Corniani and Saal, 2020).

Aβ afferents in the glabrous skin can be differentiated with respect to the size of their receptive fields and location (Johansson, 1978; Vallbo and Johansson, 1984; Johansson and Flanagan, 2009; Dahiya et al., 2010). In Vallbo and Johansson (1984) a comprehensive overview is given: corresponding to the small, accurate receptive fields, FA1 and SA1 receptors are responsible for localizing contact stimuli on the skin surface and to detect details of the surface structure at the site of contact. FA1 and SA1 units show a non-uniform distribution. They accumulate in the skin of body parts that show high tactile resolution, such as finger tips. While FA1 units are located in the papillary (close to the skin surface) SA1 units can be found at the tip of the intermediate epidermal ridges. Hence, both units are located in the very upper part of the dermis. FA2 and SA2 units have large receptive field with obscure borders. While FA2 units are found in the subcutaneous tissue SA2 units are located in the dermis but lower than FA1 and SA1 units. Vallbo et al. (1995) shows that receptive fields of hair and field afferents in hairy skin are oval or irregular in shape without orientation and larger than those of SA1 and SA2 receptors.

### 2.2. C-Tactile Afferents

C-tactile (CT) afferents are unmyelinated, low-threshold, i.e., responding strongly to light stimuli (<5 mN), mechanoreceptive nerve fibers found in the hairy skin of humans (Vallbo et al., 1999; Ackerley and Watkins, 2018). They strongly respond to slow and light tactile stimuli (Nordin, 1990; Vallbo et al., 1999; Löken et al., 2009; Morrison et al., 2011). Such stimulation characteristics are typical for caressing or stroking with a soft material, which is why it was hypothesized that CT afferents play a major role in affective touch and its social components (Löken et al., 2009; Ackerley et al., 2014; Huisman et al., 2016). This is grounded on two main observations: activations in the insular cortex of the human brain for stimulation of CT afferents (Olausson et al., 2002) and reports on maximized pleasantness when stimuli match CT-optimal characteristics.

The first observation is established by findings of neurological investigations, which have revealed that especially the contralateral posterior insula was found to respond to stroking with a soft brush (Olausson et al., 2002; McGlone et al., 2012). As the insular cortex is involved in emotional processing (Olausson et al., 2002; Leibenluft et al., 2004; Craig, 2008, 2009; McGlone et al., 2012), a social function of these nerve fibers appears obvious. This is further supported by the findings of Morrison et al. (2011), where participants watched other persons' arms being stroked and similar reactions to these purely visual stimuli were measured in the posterior insula.

The other observation, reported by multiple studies (Morrison et al., 2011; Crucianelli et al., 2013, 2018; van Stralen et al., 2014; Culbertson et al., 2018), is the stimulation being rated most pleasant by the participants when characteristics of the stimulation, e.g., stroking speed, complied with CT-optimal values. Therefore, it seems evident that CT afferents play a significant role in affective and social touch.

#### 2.2.1. Response Characteristic

The signal propagation speed of CT impulses was found to be around 0.9 m/s (Vallbo et al., 1999). With a sustained indentation, the firing rate with high-frequency impulses at initial contact attenuates within 4–5 s (Vallbo et al., 1999; Ackerley and Watkins, 2018), which indicates intermediate adaptation characteristics compared to the slowly and rapidly adapting myelinated mechanoreceptors (Olausson et al., 2010). In some CT afferents, the firing rate remain increased with irregular recurring short interspike intervals separated by much longer intervals for 30 s after an initial adaptation phase of 12 s, and then, peaked and gradually decreased with more regular firing for 40 s until cessation, which is known as delayed acceleration (Vallbo et al., 1999). Furthermore, CT fibers are prone to fatigue, i.e., the first response to a stimulus is much stronger than to a following and identical one, which can even lead to unexcitability (Nordin, 1990). After releasing skin contact, after-discharges were observed, which can last several seconds (Nordin, 1990).

#### 2.2.2. Morphology and Location

In the human body, C-tactile fibers can be found in hairy skin areas (Vallbo et al., 1999; Liu et al., 2007; Löken et al., 2009) as well as the facial area (Nordin, 1990) and glabrous hand skin (Watkins et al., 2021). Besides, forearm has often been the focus area of research conducted with regard to CT afferents due to ease of access (Vallbo et al., 1999; Wessberg et al., 2003; Löken et al., 2009; Morrison et al., 2011; Crucianelli et al., 2018; Culbertson et al., 2018).

Although there is a lack of an accurate method to estimate the distribution density of CT afferents, they were encountered as often as Aβ-afferents in previous microneurography experiences (Olausson et al., 2010). Recently, Watkins et al. (2021) suggested that CT innervation of hairy arm skin is approximately 7 times higher than that of the glabrous hand skin.

The receptive field of CT afferents was found to be circular to oval in shape without a preferred orientation (Wessberg et al., 2003). There are 1–9 responsive hot-spots distributed non-uniformly over an area from 1 to 35 mm^2^ (Wessberg et al., 2003).

We believe that considering only biological background in design of robotic devices would lead to deficient products. Therefore, these findings should be blended with the psychological requirements of humans experiencing affective touch.

## 3. Requirement Analysis

Prioritizing design requirements should depend on the application field of human-machine interfaces. Since assistive devices and prosthetics have to operate in close contact with a human, aspects of physical and cognitive human-robot interaction, and especially, psychological factors have attracted the attention of researchers (Beckerle et al., 2018). Although modalities, applications, and benefits of tactile information as a channel of communication have been a hot topic in the haptics community (Che et al., 2018; Reed et al., 2019; Ozioko et al., 2020), here we focus on the social aspects of touch as they can improve the experience of humans while interacting with devices. For the design and implementation of human-machine interfaces aiming at eliciting affective sensations, it appears promising to optimize them with regard to the particular characteristics of human C-tactile mechanoreception. As explained in the previous section, in addition to myelinated fibers, CT fibers allow humans to perceive soft and gentle stroking usually as a positive affective experience (Olausson et al., 2002). The relaxing and pleasant effects of affective touch in human-human interactions (Ditzen et al., 2008) and even human-animal interactions (Vormbrock and Grossberg, 1988) inspires the research in human-machine interaction (Eckstein et al., 2020). The technical requirements of affective touch with its effects on psychological factors are investigated in this section by extending the previous study presented by Beckerle et al. (2018).

### 3.1. Psychological Factors

Although various psychological factors affect the quality of haptic interaction, we focus on embodiment, pleasantness, and continuity as previous studies frequently related these factors to affective and social touch.

#### 3.1.1. Embodiment

Many assistive devices and systems that serve the functional substitution, such as exoskeletons and prosthetics, are designed to either support users in toilsome tasks or overcome dysfunctionalities. In either case, ensuring the harmony between acts of devices, and intentions or demands of users should be a primary goal for designers (Beckerle et al., 2017a,b). Therefore, the feeling of embodiment is a crucial psychological factor that can benefit from affective and social touch during interaction (Beckerle et al., 2018). To enable full-scale embodiment of robotic devices, bidirectional human-machine interfaces are expected to intensify dexterous control and thereby, improve user acceptance (Beckerle et al., 2019).

The potential of embodiment in robotics has led several researchers to conduct human-in-the-loop tests to evaluate embodiment (Caspar et al., 2014; Romano et al., 2015; Fröhner et al., 2018; Huynh et al., 2019; Penner et al., 2019). Motivated by the rubber hand illusion paradigm, recent studies investigate bodily self-experience and device embodiment in human-in-the-loop experiments by using either robotic hand (Caspar et al., 2014; Romano et al., 2015; Huynh et al., 2019) or robotic leg (Penner et al., 2019) as the artificial limb. Moreover, Fröhner et al. (2018) investigated how virtual limbs affect embodiment in a virtual reality environment.

Unlike the aforementioned and many other works with a psychological point of view, Crucianelli et al. (2013, 2018) incorporated the concept of affective touch to the rubber hand illusion experiment by designing an interface which is one of the few technically oriented studies considering affective aspects. They showed that slow, gentle stimuli enhance not only embodiment, but also its subfactors ownership, agency, and location. Yet, Carey et al. (2021) claimed that affective touch does not enhance subjective embodiment within the whole-body illusion but is rather body-part specific.

#### 3.1.2. Pleasantness

Pleasantness of touch can be regarded as another important modulator during social interactions (Morrison et al., 2009) and stress management (Ditzen et al., 2007; Morrison, 2016b). Therefore, psychological research puts increased emphasis on pleasant touch (Löken et al., 2009; van Stralen et al., 2014; Huisman et al., 2016; Culbertson et al., 2018). Accordingly, considering pleasantness in interface design eliciting affection appears very promising to improve user experience (van Stralen et al., 2014). Affective touch should not be confused with pleasant touch as affective touch can result in unpleasantness when stimulation characteristics are ill-adjusted. Besides stimulation characteristics, perception of (affective) touch is influenced by external factors, such as emotional expressions (Ravaja et al., 2017), olfactory environment (Croy et al., 2016) and even emotional state (Kelley and Schmeichel, 2014) and personality (Koole et al., 2014) of subjects.

#### 3.1.3. Continuity

Continuity of stimuli is an additional and relatively simple factor regarding technical implementation. While continuity is self-fulfilling in the case of continuous stimuli, such as brushing with sinusoidal motion, it can still be characterized by delay and pulse width in the case of discrete stimulation. Nevertheless, continuity should not be considered as a physical but a psychological factor since it affects the realism and pleasantness of the stimulation (Culbertson et al., 2018). Culbertson et al. (2018) stated delay and pulse width can be adjusted to maximize continuity and pleasantness.

### 3.2. Stimulation Parameters

After discussing psychological effects of affective touch, we review specific stimulation parameters which are required to mediate affective touch through human-machine interfaces from both neurophysiological and psychological perspective.

#### 3.2.1. Neurophysiological Requirements

C-tactile afferents respond highly to slow, low-pressure and soft tactile stimuli, which are similar to caressing motions (Nordin, 1990; Morrison et al., 2011; Crucianelli et al., 2018) with impulse rates in 50–100 imp/s (Vallbo et al., 1999). Since high impulse rates of CT afferents correlate positively to pleasantness (Löken et al., 2009; Ackerley et al., 2014), a range of 1–10 cm/s, with peaks at 1, 3, and 10 cm/s, is considered to be CT-optimal as the neuronal firing rate is the highest within this range (Löken et al., 2009). The relationship between the neuronal firing rate and stimulation velocity resembles an inverted parabola (Löken et al., 2009; Ackerley et al., 2014). A preference of 3 cm/s over 30 cm/s of the posterior insula was also verified using functional magnetic resonance imaging (Morrison et al., 2011). Furthermore, CT afferents are not activated by tactile stimulation at high velocities (Crucianelli et al., 2018). However, recent results indicate that they respond to vibratory stimuli in a restricted frequency range with different stimulation patterns (Wiklund Fernström et al., 2002). While CT afferents are defined as low-threshold mechanoreceptors by their activation to touch at 5 mN or less (Vallbo et al., 1999), they respond to stronger indentation forces as 0.1–0.5 N (Nordin, 1990) and 0.2–0.4 N (Löken et al., 2009). Moreover, a range of 1 N–2 N was reported to be both perceptible and comfortable on the arm (Culbertson et al., 2018). Air puffs evoke no response in CT fibers (Ackerley et al., 2014). CT fibers are not able to discriminate between pin-prick and smooth-probe stimuli as they have similar responses to both (Vallbo et al., 1999). In terms of thermal sensitivity, CT fibers show weak responses for innocuous cooling unlike heating or even noxious heat (Nordin, 1990). However, it is noteworthy that only a small subset of high-threshold CT fibers react vigorously to noxious heating (Nordin, 1990). The highest firing frequencies of C-tactile nerves occur at temperatures near skin-temperature (Ackerley et al., 2014). Besides, Ackerley et al. (2018) showed that warm touch decreases firing of CT afferents while cool touch results in lower firing rates but afterdischarge spiking.

#### 3.2.2. Psychological Requirements

Table 1 can be inspected to design human-machine interfaces considering affective touch. The table presents extracted parameters to guide interface design aiming at eliciting embodiment, pleasantness, and continuity with affective touch. The firing rate correlates positively to the perceived pleasantness ratings of the participants and, thus, a more pleasant experience (Löken et al., 2009). Apparently, 3 cm/s was tested and verified to be the most pleasant (Löken et al., 2009; Crucianelli et al., 2013, 2018; Ackerley et al., 2014; van Stralen et al., 2014). Beyond the previously mentioned velocity range of 1–10 cm/s, Culbertson et al. (2018) reported that a velocity of 13.5 cm/s was “interestingly” the slowest speed that was perceived as pleasant. It should be kept in mind that Culbertson et al. (2018) designed their interface to evaluate linear lateral motion on an arm by applying only normal forces for sequential indentation with an array of voice coils. They stated that slower speed stroking causes unpleasant and creepy feelings, which explains this “interesting” result by Culbertson et al. (2018). The discrete nature of the work also explains why researchers did not consider continuity as a factor, and pulse width and delay as parameters since they applied stimuli with continuous motion of a stimulator, except Culbertson et al. (2018). Embodiment and pleasantness are maximized during the rubber hand illusion when the stimulus is synchronous rather than asynchronous (Crucianelli et al., 2013, 2018; van Stralen et al., 2014). Additionally, pleasantness and continuity feelings are improved with vibration as long as vibration applied has low intensity (Huisman et al., 2016; Culbertson et al., 2018). Finally, softer materials make the touch more pleasant according to van Stralen et al. (2014).

**Table 1:** Optimal parameters of stimulation to maximize psychological factors mentioned in section 3.1.

	**Embodiment**	**Pleasantness**	**Continuity**
Essick et al. (1999)	-	Velocity: 5 cm/s Material hardness: soft (velvet, cotton)	-
Löken et al. (2009)	-	Velocity: 1, 3, 10 cm/s	-
Crucianelli et al. (2013)	Velocity: 3 cm/s		
Synchronicity: synchronous	Velocity: 3 cm/s	-	
van Stralen et al. (2014)	Velocity: 9 cm/s	Velocity: 3 cm/s Synchronicity: synchronous Material hardness: soft (brush)	-
Huisman et al. (2016)	-	Velocity: 6.41 cm/s Vibration: low intensity (Amplitude: 0.9 g, Frequency: 140 Hz)	-
Crucianelli et al. (2018)	Velocity: 9 cm/s	Velocity: 3 cm/s Synchronicity: synchronous	-
Culbertson et al. (2018)	-	Velocity: 13.5 cm/s Vibration: low amplitude Delay: low (12.5%) Pulse width: long (800 ms)	Vibration: low amplitude Delay: low (12.5%) Pulse width: long (800 ms)

*The table presents the key findings of related research with a focus on psychological factors*.

## 4. Design Recommendations

From the detailed requirement analysis provided above, design specifications can be inferred to select and dimension components, e.g., actuators and sensors to meet the neurophysiological and psychological requirements. Besides the stimulation parameters mentioned there, a design should also align the haptic resolution of actuators and sensors with the physiological features of human sense of touch (Kern and Hatzfeld, 2014). Since haptic devices can be based on tactile stimulation, tactile sensing, or a combination of both principles as in telemanipulation, resolution of both actuators and sensors is expected to match physiological features of humans. While actuators should be as accurate as human perception can resolve, sensors should be as sensitive as human skin so that bidirectional haptic information can be transmitted (Kern and Hatzfeld, 2014).

While resolution requirements apply to any haptic system, they strongly depend on the location of stimulation on the human body and, thus, vary with the respective mechanoreceptor population, skin properties, and spatial acuity (Dahiya et al., 2010). So far, there is limited knowledge and research about interfaces to excite CT afferents. One of the first attempts of a haptic interface directly addressing social touch was “Tactile Sleeve for Social Touch” by Huisman et al. (2013). They designed a sleeve consisting of an input layer with force-sensing resistors and an output layer with vibration motors in the shape of a 4 by 3 grid. Sensors were designed to detect forces around 0.4 N, while vibration stimulation was controlled to proportionally code the applied force. A similar interface with cylindrical vibration motors instead of coin-type ones was used to investigate how velocity and intensity of vibrotactile stimuli affect pleasantness (Huisman et al., 2016). Another example of vibrotactile stimulation is presented by Raisamo et al. (2013) as they tested three stimulation patterns to determine their effect on pleasantness and continuity: saltation, modulation, and hybrid. Saltation provides separate pulses, modulation uses dynamic transition of amplitude between the actuators, and hybrid combines separate pulses of saltation to modulation. The modulation method was rated more continuous and pleasant, and stimuli rated as more continuous were also rated more pleasant and more relaxing (Raisamo et al., 2013). The aforementioned studies showed positive effects of low intensity vibrotactile stimuli as we stated in Table 1. Essick et al. (2010) implemented a rotary tactile stimulator to investigate textured-surface stimuli along different body site with controlled force and velocity. Instead of designing an interface based on continuous stimuli, Culbertson et al. (2018) presented a novel approach by lining up an array of voice coil actuators in a sleeve to mimic linear lateral motion like a caress. Actuators consistently applied 1–2 N of normal force while effects of delay and pulse width during sequential excitation on pleasantness and continuity were evaluated positively.

Although vibrotactile stimulation is not the only way to elicit pleasant sensations, it is frequently preferred in previous designs possibly due to ease of use and control of vibration motors. We believe that there is a lack of sophisticated designs that aim CT fiber excitation based upon requirements explained in this review, especially velocity and force requirements. Although the aforementioned designs apply, in addition to normal forces, lateral forces via vibration motors or voice coil arrays, we think that measuring and controlling shear forces can significantly improve performance of interfaces as highlighted by Beckerle et al. (2018). An array or even a matrix of linear actuators with position control of indentation can also be composed to investigate effects of stimulation patterns and indentation length on affective touch. A similar approach has been validated for a haptic interface using shape memory alloys (Hamdan et al., 2019; Muthukumarana et al., 2020) and can be adapted for affective touch. The research can be extended by modeling the impedance characteristics of human skin. Variable stiffness actuators can be investigated to implement an interface based on stiffness or impedance measurement rather than normal forces if current designs can be dimensionally minimized. Alternatively, twisted and coiled polymer actuators on a silicon skin present promising results for soft haptic interaction (Chossat et al., 2019). Along with mechanical enhancements, the response of CT fibers to thermal stimulation is still undiscovered as experiments to date have been performed at ambient temperature. Based on findings regarding responses of C-tactile afferents to thermal stimuli (Nordin, 1990; Ackerley et al., 2014, 2018), it would be benign to consider effects of different temperatures of touch in interface design by adapting mechanical and thermal stimulators, such as pneumatic haptic display (Lee et al., 2021).

## 5. Conclusion

Slow and gentle stimulation of human skin with high CT fiber population, such as hairy areas of the forearm, results in affective experiences going beyond tactile information transfer. Human-machine interfaces exciting CT afferents can be used to elicit affective reactions and thereby enhance embodiment of assistive devices. Beyond embodiment, increasing pleasantness and realism of interactions by mediating social touch can boost the acceptance levels and performance of assistive devices. Yet, this requires designers to optimize actuators and sensors with respect to the specific stimulation parameters of CT afferents which are compiled and structured in this review. In addition, one should note that different understandings of affective touch, i.e., pleasant touch and social touch, might change the design perspective.

Current shortcomings and possible improvements comprise the inclusion of lateral forces since affective touch is usually achieved by caressing or stroking hairy skin. However, there is not enough data from measurements for numerical analysis of lateral force requirements of affective touch: measuring shear forces can change the perspective of designers during actuator selection to create stronger emotional responses. Moreover, all interfaces designed so far are only suitable for laboratory use. Wearable interfaces for tests in daily life application appear commercially interesting, but also bear an exceptional potential to fundamentally investigate the effectuality of multisensory affective touch effects and interfaces in the field where multisensory stimuli exist unlike the laboratory environment.

## Author Contributions

MC, DN, EK, and PB conceptualized the article and coordinated its development. DN and MC analyzed the requirements. All authors provided perspectives and references as well as discussed and revised the manuscript.

## Conflict of Interest

The authors declare that the research was conducted in the absence of any commercial or financial relationships that could be construed as a potential conflict of interest.
